# Transrectal Absorber Guide Raster‐Scanning Optoacoustic Mesoscopy for Label‐Free In Vivo Assessment of Colitis

**DOI:** 10.1002/advs.202300564

**Published:** 2023-04-21

**Authors:** Adrian Buehler, Emma Brown, Lars‐Philip Paulus, Markus Eckstein, Oana‐Maria Thoma, Mariam‐Eleni Oraiopoulou, Ulrich Rother, André Hoerning, Arndt Hartmann, Markus F. Neurath, Joachim Woelfle, Oliver Friedrich, Maximilian J. Waldner, Ferdinand Knieling, Sarah E. Bohndiek, Adrian P. Regensburger

**Affiliations:** ^1^ Department of Pediatrics and Adolescent Medicine University Hospital Erlangen Friedrich‐Alexander‐Universität (FAU) Erlangen‐Nürnberg 91054 Erlangen Germany; ^2^ Department of Physics and Cancer Research UK Cambridge Institute University of Cambridge Cambridge CB2 0RE UK; ^3^ Institute of Pathology Friedrich‐Alexander‐Universität (FAU) Erlangen‐Nürnberg 91054 Erlangen Germany; ^4^ Department of Medicine 1 University Hospital Erlangen Friedrich‐Alexander‐Universität (FAU) Erlangen‐Nürnberg 91052 Erlangen Germany; ^5^ Department of Vascular Surgery University Hospital Erlangen Friedrich‐Alexander‐Universität (FAU) Erlangen‐Nürnberg 91054 Erlangen Germany; ^6^ Institute of Medical Biotechnology Department of Chemical and Biological Engineering Friedrich‐Alexander‐Universität (FAU) Erlangen‐Nürnberg 91052 Erlangen Germany

**Keywords:** dextran sodium sulfate induced colitis, inflammatory bowel disease, murine acute colitis, optoacoustic imaging, photoacoustic imaging, rasterscanning optoacoustic mesoscopy

## Abstract

Optoacoustic imaging (OAI) enables microscale imaging of endogenous chromophores such as hemoglobin at significantly higher penetration depths compared to other optical imaging technologies. Raster‐scanning optoacoustic mesoscopy (RSOM) has recently been shown to identify superficial microvascular changes associated with human skin pathologies. In animal models, the imaging depth afforded by RSOM can enable entirely new capabilities for noninvasive imaging of vascular structures in the gastrointestinal tract, but exact localization of intra‐abdominal organs is still elusive. Herein the development and application of a novel transrectal absorber guide for RSOM (TAG‐RSOM) is presented to enable accurate transabdominal localization and assessment of colonic vascular networks in vivo. The potential of TAG‐RSOM is demonstrated through application during mild and severe acute colitis in mice. TAG‐RSOM enables visualization of transmural vascular networks, with changes in colon wall thickness, blood volume, and OAI signal intensities corresponding to colitis‐associated inflammatory changes. These findings suggest TAG‐RSOM can provide a novel monitoring tool in preclinical IBD models, refining animal procedures and underlines the capabilities of such technologies to address inflammatory bowel diseases in humans.

## Graphical abstract

Optoacoustic imaging uses laser light excitation and ultrasound detection for deep tissue imaging. Transrectal absorber guide raster‐scanning optoacoustic mesoscopy (TAG‐RSOM) is created to achieve microscopic decoding of transmural vascular networks during inflammation in murine colitis in vivo. These findings underline the capabilities of optoacoustic technologies to address inflammatory bowel diseases in humans.

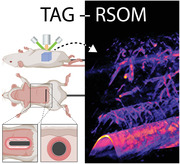

## 1 Introduction

Inflammatory bowel diseases (IBD) − comprising two main entities, namely ulcerative colitis and Crohn's disease − are a global burden to patients and healthcare systems with a steadily rising incidence affecting at least 0.5% of the population.^[^ ^1^ ^]^ While novel drugs are continuously developed,^[^ ^2^ ^]^ imaging biomarkers for the evaluation of mucosal healing – the ultimate goal in the treatment of IBD patients – are still controversially discussed.^[^ ^3^ ^]^ Until then, white light endoscopy followed by histopathology remains the reference standard to assess disease remission.^[^ ^4^ ^]^ Advanced endoscopic optical technologies can provide cellular resolution for intestinal imaging but are limited to the mucosal surface^[^ ^5^ ^]^ while tomographic imaging technologies such as computed tomography (CT)^[^ ^6^ ^]^ and magnetic resonance imaging (MRI)^[^ ^7^ ^]^ access deep‐tissue but are limited in resolution to 0.1 – 1 mm scale in patients.

Optoacoustic imaging (OAI) is an emerging non‐invasive, non‐ionizing clinical imaging modality that is ideally suited to address this unmet need to better monitor inflammatory disease severity and remission. OAI uses pulsed laser light to generate acoustic waves that can be detected at the tissue surface,^[^ ^8^ ^]^ which enables a trade‐off between penetration depth and spatial resolution. In tomographic mode, OAI can achieve deep‐tissue imaging up to several centimetres with a resolution typically ≈100 µm, while implementation in mesoscopy mode limits penetration depth to a few millimetres but increases resolution. For example, raster‐scanning optoacoustic mesoscopy (RSOM) can directly visualize vascular networks with a resolution of up to 5 – 10 µm axially and 20 – 40 µm laterally, and a penetration depth of up to 3 mm.^[^ ^9^ ^]^

OAI has shown promise in early clinical trials in cardiovascular diseases,^[^ ^10^ ^]^ evaluation of muscle wasting,^[^ ^11^ ^]^ and monitoring of inflammatory diseases,^[^ ^12^ ^]^ including IBD.^[^ ^13^ ^]^ Experimental studies of tomographic OAI in mice demonstrated the quantification of inflammation,^[^ ^14^ ^]^ and identification of intestinal fibrosis,^[^ ^15^ ^]^ too. Higher resolution imaging of vascular networks with RSOM has been shown in ex vivo imaging of intestinal tumors and murine colitis^[^ ^16^ ^]^ and in vivo models of skin and xenograft tumors.^[^ ^17^ ^]^ In early clinical studies, RSOM has been used to image superficial human skin pathologies.^[^ ^18^ ^]^ Moreover, RSOM technology is now being deployed in endoscopic systems.^[^ ^5^, ^19^ ^]^

We therefore hypothesized that RSOM vascular biomarkers could be applied for precise assessment of IBD through deep‐tissue imaging of inflammatory biomarkers. At present, accurate identification, assessment, and evaluation of the transmural vascular network as an imaging biomarker for mucosal healing is still elusive. To study the potential of RSOM in this setting, we developed a new approach, transrectal absorber guide RSOM (TAG‐RSOM), which allowed us to define and evaluate RSOM vascular biomarkers for IBD in a controlled mouse‐model setting of mild and severe acute colitis. Using our TAG‐RSOM approach, we were able to accurately monitor disease severity, suggesting positive indications for the future translational potential of RSOM in IBD.

## 2 Results

### 2.1 Development of a Transrectal Absorber Guide (TAG) for Precise RSOM Imaging of Colonic Vasculature

**Figure 1:**
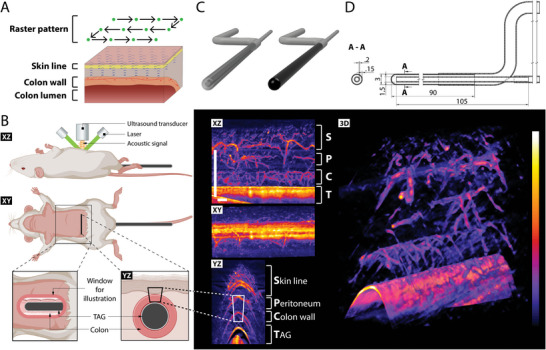
Transrectal Absorber Guide Raster‐Scanning Optoacoustic Mesoscopy (TAG‐RSOM) for imaging vascular networks of the colon. A) Raster scanning over a field of view of 12 × 12 × 3 mm^3^ with a lateral resolution of 40 µm, an axial resolution of 10 µm, and an imaging depth up to 3 mm depth allows transabdominal visualization of colon vasculature. B) Schematic illustration of TAG‐RSOM. Laser light with a wavelength of 532 nm illuminates the tissue. A single‐element transducer receives the emitted pressure waves. (white panel, created with Biorender). Exemplary image of the murine colonic vasculature by TAG‐RSOM containing ink. TAG allows clear separation of skin and peritoneal vessels from the adjoining colon wall vascular network (black panel). S = skin line, P = peritoneum, C = colon wall, and T = TAG. C) Rendering of the TAG. Left panel: TAG filled with water (transparent); Right panel: TAG filled with ink (black). See also Video S1 (Supporting Information) for technical realization. Created with Fusion 360 software (Version 2.0.14567, Autodesk Inc., San Rafael, USA). D) Technical drawing of the TAG. The TAG contains two interleaved plastic tubes that allow an exchange of fluids. Therefore, changing of contrast agents is possible during in vivo imaging without displacing the animal. Length is in mm.

Transabdominal in vivo RSOM was performed in a field‐of‐view up to 12 × 12 × 3 mm^3^ (**Figure** **1**A,B). Raster‐scanning allowed the formation of images of vascular networks at a depth of up to 3 mm based on the absorption of light by hemoglobin. While superficial skin vasculature was easy to delineate, intra‐abdominal colonic vasculature had to be differentiated from the peritoneal vasculature (Figure 1B). Therefore, we developed the TAG with a liquid chamber which was filled with either ink (“ink‐TAG”) or water (“water‐TAG”) (Figure 1C,D; Video S1, Supporting Information); ink is a highly light‐absorbing substance that provides a clear outline of the colon wall in the RSOM images; water was used to ensure exclusion of imaging artifacts caused by the ink. Exact co‐registration of both scans for advanced OAI analysis was enabled by maintaining the positioning of the mouse unchanged during imaging. Subsequent image segmentation enabled the separation of the colon wall vasculature from all other adjacent tissue (Figure S1, Supporting Information) in high resolution (Figure S2, Supporting Information). Colon wall thickness, blood volume, and OAI signal intensity were used as imaging biomarkers.

### 2.2 TAG‐RSOM Imaging Shows Sensitive Detection of Mild Acute Colitis (One Cycle of DSS)

**Figure 2:**
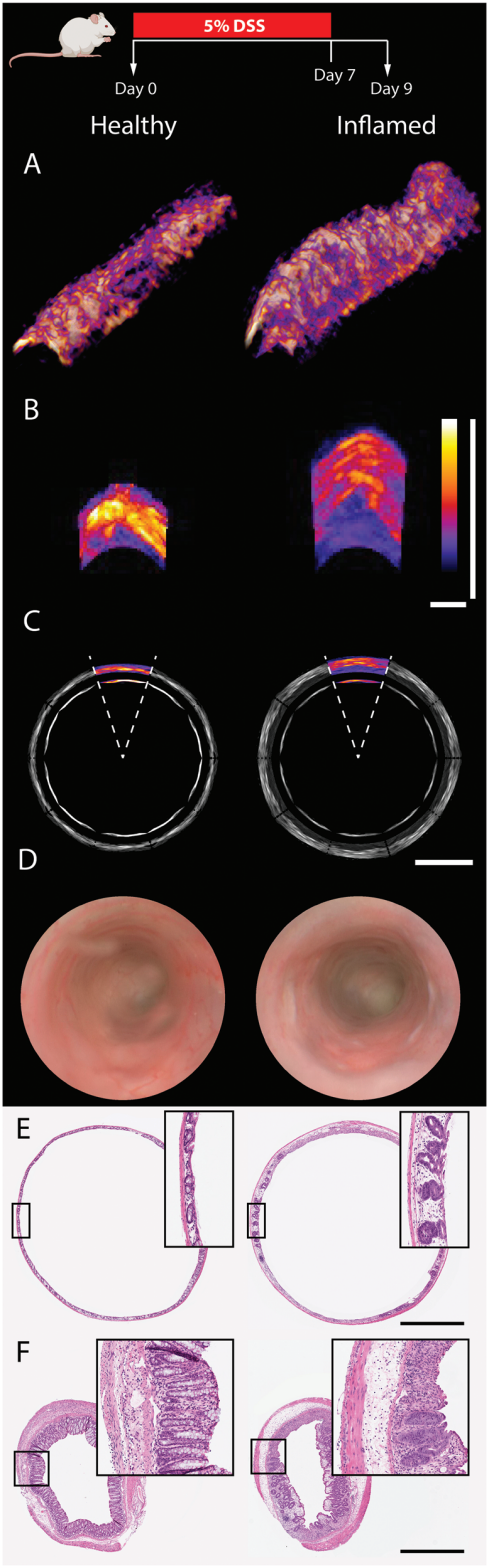
Illustration of cross sectional TAG‐RSOM imaging of acute colitis. RSOM, endoscopic and histological images of one mouse before and after induction of acute colitis by 5% DSS. A) 3D visualization of RSOM images of the colonic vasculature revealed colon wall thickening by more strongly perfused vascular networks. B) Transverse maximum intensity projections illustrate the increase in colon wall thickness. Both scale bars represent 400 µm. C) Synthetic illustrations of multiplied RSOM images from B mimic the impression of the respective endoscopy and histopathology while underlining the limited field of view of ± 18° relative to the X‐axis (TAG‐axis). The scale bar represents 1000 µm. D) Endoscopic images of the colon with healthy mucosa (left), and inflamed mucosa with fibrin deposition on the right after DSS administration. E,F) Histopathological images from the TAG stretched E) and standard F) H&E sections of the colon showing wall thickening, inflammatory cell infiltrates, and mucosal ulcerations after DSS administration. Scale bars represent 1000 µm.

TAG‐RSOM was able to visualize transmural vascular network changes regarding increased colon wall thickness and vascularity after the onset of acute colitis (**Figure** **2**A,B). Furthermore, colon wall transformation with increased irregularity was seen over the colon length, however, the field‐of‐view is limited to the upper surface of the colon (Figure 2C). Acute colitis detected by RSOM was in line with increased mucosal damage seen by endoscopy and histopathology (Figure 2D–F).

**Figure 3:**
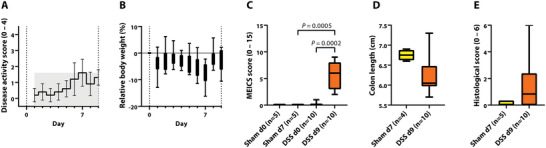
Disease assessment of mild colitis. Mice were either treated as sham control (*n* = 5) or with 5% DSS (*n* = 10) over seven days to induce acute colitis. A,B) Daily monitoring of disease activity score and relative body weight as signs of the development of acute colitis. C) Verification of acute colitis by endoscopic scoring. MEICS = murine endoscopic index of colitis severity. D,E) Verification of acute colitis by colon length shortening and histological scoring. DSS = dextran sulfate sodium, d = day; *P* < 0.05 was considered statistically significant.

The presence of acute colitis was proven by worsening of disease activity score, a decrease in relative body weight at day seven (**Figure** **3**A,B), and endoscopic disease severity scoring (0.20 ± 0.42 vs 5.70 ± 2.71, *P* = 0.0002) (Figure 3C). No statistically significant changes regarding colon length shortening (6.75 ± 0.13 cm vs 6.24 ± 0.47 cm, *P* = 0.0571) or histologic scoring (0.13 ± 0.18 vs 1.55 ± 2.17, *P* = 0.17) were observed post mortem (Figure 3D,E).

**Figure 4:**
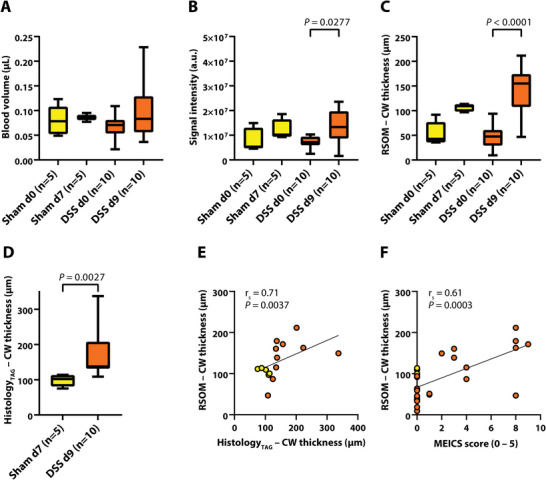
RSOM imaging of mild colitis. The data displayed in this figure were generated using ink as a contrast agent in TAG‐RSOM. For data analysis based on water‐TAG see Figure S3 (Supporting Information). A–C) RSOM derived blood volume A), and signal intensities B) in sham control and mild colitis on day zero and seven/nine.; *P* < 0.05 was considered statistically significant. D–F) RSOM C) and histology D) derived colon wall thickness with respective correlation E,F). DSS = dextran sulfate sodium, d = day, TAG = transrectal absorber guide, CW = colon wall, r_s_ = Spearman correlation coefficient; *P* < 0.05 was considered statistically significant.

Total blood volume measured by RSOM increased after induction of acute colitis; however, no statistically significant difference was found (all *P* > 0.05, **Figure** **4**A). OAI signal intensity, however, significantly increased in mice with mild acute colitis (day 0 vs 9: 7.3 × 10^6^ ± 2.3 × 10^6^ a.u. vs 1.3 × 10^7^ ± 6.8 × 10^6^ a.u., *P* = 0.0277) (Figure 4B). Furthermore, an increase in OAI derived colon wall thickness (46.2 ± 23.91 µm vs 142.3 ± 48.1 µm, *P* < 0.0001) (Figure 4C) could also be observed in measurements compared to histological H&E staining (sham day 7 vs DSS day 9: 98.0 ± 16.3 µm vs 169.7 ± 66.7 µm, *P* = 0.0027) (Figure 4D). TAG‐RSOM measurements positively correlated with histological thickness measurements (r_s_ = 0.71, *P* = 0.0037) and endoscopic disease assessment (r_s_ = 0.61, *P* = 0.0003) (Figure 4E,F). TAG‐RSOM data were not affected by the presence of either ink or water in the TAG (see Figure S3, Supporting Information); all data presented in the main text are from ink TAG images.

### 2.3 TAG‐RSOM can be Applied for Longitudinal Monitoring of Severe Acute Colitis (Two Cycles of DSS)

**Figure 5:**
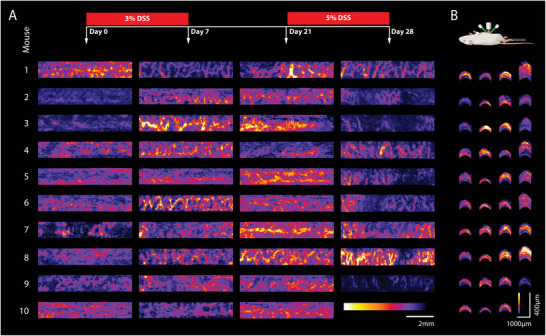
Longitudinal RSOM imaging of acute colitis. Mice were treated with two cycles of DSS to induce increasing acute colitis over 28 days. The two cycles of DSS, one with 3% and one with 5%, were discontinued by a recovery phase of 14 days in between. All mice were imaged with TAG‐RSOM before and after each cycle of DSS. Here, we present RSOM maximum intensity projections of the colon segments of each mouse in frontal A) and transverse B) planes.

TAG‐RSOM allowed for repeated anatomical identification of the colonic vasculature over the time course of the experiment, revealing longitudinal visual changes in vascular patterns. In particular, after the second cycle of DSS, the vascular network had a patchy appearance with clusters of thickened or recessed vessels (**Figure** **5**A). Furthermore, in the transverse plane, thickening of the colonic wall was seen over the course of the experiment (Figure 5B).

**Figure 6:**
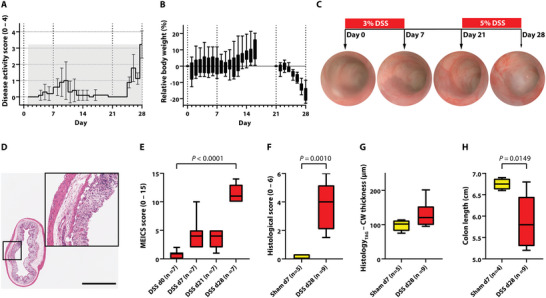
Disease assessment of severe colitis. Severe acute colitis was induced in mice by administering two cycles of treatment: one with 3% DSS and one with 5% DSS over seven days each, with a 14‐day recovery phase in between cycles. A,B) Daily monitoring of disease activity score A) and relative body weight B) during the experiment as signs of the development of acute colitis. C,D) Exemplary endoscopic C) and histological D) images of the developing acute colitis during the experiment. E–H) Verification of acute colitis by endoscopic E) and histological F) scoring, histological colon wall thickness measurement G), and colon length shortening H). DSS = dextran sulfate sodium, d = day, TAG = transrectal absorber guide, CW = colon wall, MEICS = murine endoscopic index of colitis severity; *P* < 0.05 was considered statistically significant. Scale bar represents 1000 µm.

Our experiments with one cycle of low‐dose (3%) DSS induced only very subtle colitis, as reflected by barely altered disease activity score and no significant decrease in relative body weight. Furthermore, recovery was seen by the normalization of the aforementioned scores (**Figure** **6**A,B). However, the second cycle of high‐dose (5%) DSS led to pronounced acute colitis, manifested by markedly increased disease activity score and loss of body weight (Figure 6A,B). Acute colitis was confirmed by endoscopy, histology, and colon length shortening (Figure 6C–H). While the endoscopic score was non‐significantly increased after one cycle of low‐dose DSS (day0 vs d7: 0.71 ± 0.76 vs 4.29 ± 2.75, *P* = 0.1153), a significant deterioration of health status was observed after the second cycle of high‐dose DSS (day 0 vs 28: 0.71 ± 0.76 vs 11.71 ± 1.60, *P* < 0.0001) (Figure 6E).

**Figure 7:**
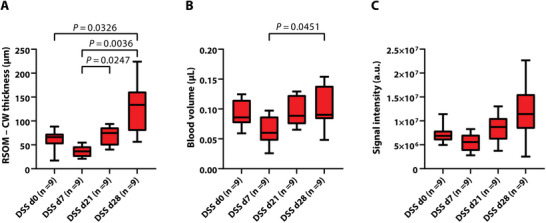
Longitudinal TAG‐RSOM imaging of severe colitis. The data displayed in this figure were generated using ink as a contrast agent in TAG‐RSOM. For data analysis based on water‐TAG see Figure S3 (Supporting Information). A–C) RSOM colon wall thickness A), derived blood volume B), and signal intensities C) in the severe colitis group. DSS = dextran sulfate sodium, d = day, CW = colon wall; *P* < 0.05 was considered statistically significant.

Quantification of TAG‐RSOM images confirmed the observed findings. While one cycle of DSS did not increase colon wall thickness, a distinct increase was seen after the second cycle in severe colitis (day 0 vs 28: 60.90 ± 20.67 µm vs 127.3 ± 53.65 µm, *P* = 0.0326) (**Figure** **7**A). TAG‐RSOM derived blood volume increased over the course of the experiment, with a significant difference between day 7 and 28 (0.07 ± 0.02 µL vs 0.11 ± 0.04 µL, *P* = 0.0451) (Figure 7B). In addition, RSOM signal intensity increased, similarly to the result found in mild colitis, although non‐significant (all *P* > 0.05) (Figure 7C).

## 3 Discussion

In this study, we developed and applied TAG‐RSOM to allow for clear identification of the vascular network of the colon. We provide evidence for the precise localization and visualization of transmural colon wall vasculature using our in vivo label‐free TAG‐RSOM approach in murine models of DSS‐induced colitis. Advanced image data processing enabled the segmentation of individual colonic layers for assessment of inflammation in the colon wall.

TAG‐RSOM derived colon wall thickness correlated well with endoscopic and histological disease assessment, showing good morphological agreement. Our findings of increased colon wall thickness match observations of other preclinical imaging modalities.^[^ ^20^ ^]^ Studies using small animal MRI^[^ ^7^, ^20^ ^]^ attributed the colon wall thickening to increased immune cell infiltration, oedema, and hypervascularization. Moreover, TAG‐RSOM revealed increased signal intensity in markedly inflamed colon segments after two cycles of DSS administration. The severity of colitis measured by TAG‐RSOM was verified by weight loss, increased disease severity, endoscopic, and histological scores and macroscopic organ shortening. Similar, but less pronounced results were found in mice after one cycle of high‐dose (5%) DSS while by contrast, one cycle of lower‐dose (3%) DSS neither altered health status nor RSOM scoring. Our findings align with prior studies conducted in lower‐resolution tomography systems, which showed that OAI hemoglobin signal intensity correlated well with other measures for disease activity^[^ ^13^, ^14^ ^]^ in both intestinal inflammation in enterotoxigenic *Bacteroides fragilis*‐mediated colitis in mice^[^ ^14^ ^]^ and in IBD patients.^[^ ^13^ ^]^

In contrast to tomographic OAI approaches, the increased resolution of RSOM not only allows signal quantification but also a detailed mapping of the transmural vascular network patterns. In our study, mice with severe colitis showed patchy clusters of thickened or recessed vessels. As has been previously reported,^[^ ^21^ ^]^ microcirculation is dynamically altered during the course of chemically‐induced murine colitis. Such changes can be attributed to inflammation, altered vascular permeability, and vessel thickness. Imaging technologies like fluorescent confocal laser endomicroscopy in combination with dynamic contrast‐enhanced MRI described similar patchy clusters as differences between the well‐structured honeycomb in healthy mice compared to distorted patterns of microvasculature and dilaed large vessels in acute colitis.^[^ ^22^ ^]^ Nonetheless, confocal laser endomicroscopy requires the addition of contrast agents and is limited to superficial inspection of tissues.^[^ ^23^ ^]^

TAG‐RSOM provides the unprecedented ability to directly image these changes of the transmural vascular network, which was previously only accessible to ex vivo microscopy technologies.^[^ ^24^ ^]^ TAG‐RSOM was developed for preclinical studies of acute colitis in mice. Next, angiogenesis – the hallmark of cancer development – could be addressed in models of colitis‐associated cancer in accordance with RSOM studies on skin and xenograft tumor models.^[^ ^17^ ^]^ With the knowledge gained from such studies, OAI technologies integrated into endoscopic systems might enable the determination of these angiogenic biomarkers in vivo in patients.^[^ ^5^, ^19^ ^]^

This study has several limitations. TAG‐RSOM was performed using a single wavelength depicting mainly, but not exclusively, signals from hemoglobin molecules. By using RSOM at multiple wavelengths, other endogenous chromophores such as oxygenated/deoxygenated hemoglobin, and other absorbers might be differentiated.^[^ ^12^, ^25^ ^]^ Furthermore, in our approach, different concentrations of DSS were applied to mimic mild and severe colitis. Future studies might consider using larger, more homogenously composed groups in order to improve the reliability and generalizability of the results using RSOM. However, both experiments showed overall consistent imaging results with compelling visualization of transmural vascular networks.

In summary, we demonstrated that TAG‐RSOM is a unique technology for in vivo transmural visualization of the vascular network of the murine colon. Such detailed information about murine colitis at microscopic resolution might help to decipher novel pathology pathways. Furthermore, our approach supports the development of such technologies to find a translational path. The development of endoscopic optoacoustic mesoscopy imaging systems for application in the gastrointestinal tract would provide direct imaging of the human colon wall vasculature at comparable resolution to this study, while the future use of contrast agents could help outline the colon during optoacoustic tomography examinations.^[^ ^5^, ^19^, ^26^ ^]^

## 4 Experimental Section

### Animals Protocol and Handling

All animal procedures were approved by the Animal Welfare and Ethical Review Board at Cancer Research UK Cambridge Institute (project licence PE12C2B96, user licence I24947753), and issued under the United Kingdom Animals (Scientific Procedures) Act, 1986. All animals underwent daily clinical examination by disease severity scoring (0 − 4, based on relative body weight, stool consistency, and hemoccult), and body weight measurement.

Mild and severe colitis were induced by oral administration of water‐dissolved dextran sulfate sodium (DSS)^[^ ^27^ ^]^ (MP Biomedicals, Irvine, CA, USA) in different concentrations to female mice (Charles River, UK).

For induction of mild colitis, mice (*n* = 10) were supplied with 5% DSS in drinking water for seven days, followed by two days of water. Imaging was performed before and two days after DSS administration. The mice were then sacrificed on day nine.

For induction of severe colitis, mice (*n* = 10) were supplied with two seven‐day cycles of DSS, the first with 3% DSS and the second with 5% DSS with and 14 days interval in between. Imaging was performed on days 0, 7, 21, and 28. The mice were then sacrificed on day 28. To test the experimental setup for nonspecific effects, sham controls (*n* = 5) underwent the same imaging protocol on days zero and seven and were then sacrificed on day 7. All mice were aged between 49 and 55 days, had body weights ranging from 15.1 − 21.7 g, and were kept in groups of five during all studies. Detailed information for each subject can be found in Table S1 (Supporting Information).

### RSOM Data Acquisition

Imaging was performed by a commercial RSOM imaging system (RSOM explorer P50, iThera Medical GmbH, Munich, Germany). A single wavelength of 532 nm was used to generate thermoelastic expansion of molecules. The emitted sound waves were detected by a single transducer with a center frequency of 50 MHz. Raster scanning allows imaging over a field of 12 × 12 × 3 mm and could be adjusted individually with a lateral resolution of 40 µm and axial resolution of 10 µm. The acquisition parameters for the light source were set to 85% with a pulse repetition rate of 1000 Hz.

Mice were initially anesthetized with 4% isoflurane in 50% oxygen and 50% medical air. Throughout the subsequent imaging, the respiratory rate was maintained at a constant rate of 70 – 80 bpm by reducing the isoflurane to ≈1 – 2%. The fur in the abdominal region was removed first by shearing and then by applying hair removal cream for 60 s. Feces were flushed out by squirting sterile saline at 37 °C into the rectum of the mouse with a pipette to allow insertion of the TAG and subsequent colonoscopy. Body temperature was maintained by placing a heating pad under the mice, and the eyes were protected from drying by applying eye hydrogel.

### TAG

The transrectal absorber guide (TAG) was designed to enable accurate identification of the colon by providing a high‐contrast surface as a reference point beneath the colon wall. Depending on the experiment, a contrast agent (“ink‐TAG”, black drawing ink, Creative Art Products Ltd., Middlewich, UK) can be turned on and off (“water‐TAG”) (Figure 1C) without changing the positioning by flowing the respective fluid through the TAG. The flow system is achieved by inserting a thinner glass capillary tube (Radiometer Medical ApS, Brønshøj, Denmark) into a transparent polypropylene straw (Herald Plastic Ltd., Barking, UK) to ensure fluid flow (Figure 1D). The outer straw and inner tube were sealed with hot melt adhesive (PN: T6219025, RS Components, Corby, UK). A glass capillary is chosen as the inner tube as it provides the TAG with the rigidity necessary to displace the colon toward the skin and into the range of RSOM imaging depth.

### Endoscopy Device and Scoring

All animals were examined in vivo by a small animal colonoscopy system (Karl Storz, Tuttlingen, Germany). Disease severity was assessed using the murine endoscopic index of colitis severity (MEICS) according to Becker et al.,^[^ ^28^ ^]^ which is based on the thickening of the colon, changes in vascular pattern, visibility of fibrin, the granularity of the mucosal surface and stool consistency.^[^ ^28^ ^]^ Sessions were recorded and validated by a second observer (FK).

### Histology

A colon segment ≈3 – 4.5 cm from the anus was excised and divided into two equally sized pieces, each ≈4 mm in length. One piece was threaded onto a polymer tube with the TAG corresponding diameter of 3 mm. All samples were fixed in 10% formalin, embedded in paraffin, sectioned, and stained with H&E according to standard protocols, performed by the CRUK Cambridge Institute Histopathology Core Facility. Sections were scanned at 20x magnification using an Aperio ScanScope (Leica Biosystems, Milton Keynes, UK) and transferred in digital form to the institutional data servers of the University Hospital Erlangen. A board‐certified pathologist (ME) scored each H&E section in a blinded fashion according to Erben et al.^[^ ^29^ ^]^ using QuPath software (Version 0.3.2).^[^ ^30^ ^]^ For comparison with RSOM, colon wall thickness was measured in stretched tissue state using ImageScope software (Version v12.4.6.5003, Leica Biosystems, Milton Keynes, UK).

### RSOM Data Processing Protocol

RSOM images were reconstructed using a back projection algorithm with voxel size 20 × 20 × 4 µm^3^ (X, Y, Z), and motion correction was performed by the commercially supplied software (viewRSOM Version v2.3.5.2, iThera Medical GmbH, Munich, Germany). Thereafter, images were further processed with Fiji software (ImageJ version 1.53q, National Institute of Health, USA). RSOM images derived from ink‐TAG were used to identify regions of interest (ROI) and transformation operations which were then transferred to co‐registered RSOM images derived from the water‐TAG (Figure S1, Supporting Information).

To identify the same ROI in both ink‐TAG and water‐TAG scans, the image of the central part of the colon was cut to a dimension of 6 × 3 × 2 mm^3^ which corresponded to the largest colon volume that contained the imaging data across all studies. Subsequently, the volumes were rotated and the TAG was aligned along the X‐axis to automate further evaluation and to perform subsequent thickness measurements of the intestinal wall. In detail, the intersections of the TAG with the XY‐plane furthest from each other with respect to the Z‐axis – limited by the edges of the image – were defined, the rotation angle was calculated by triangulation, and the volumes were transformed by Quintic B‐Spline interpolation (ImageJ Plug‐in JTransform).^[^ ^31^ ^]^ Co‐registration (ImageJ Plug‐in Fijiyama, Handsome Honeysuckle)^[^ ^32^ ^]^ was applied to align the corresponding scans with water‐ and ink‐filled TAG.

Uplifting of the intestinal wall toward the skin layer to remain within the maximum imaging depth of RSOM causes a slight bending of the TAG. To correct this, the deformation was marked frame‐wise in the YZ‐plane along the X‐axis. The interface of the TAG's lumen and its outer polymer cylinder were used as reference points for this purpose. Linear interpolation was used to calculate new YZ‐coordinates and correct for deviations. After pre‐processing, the dimension of the analysis volume was 5480 × 1600 × 520 µm^3^.

*Thickness Measurement*: The RSOM thickness of the colon wall was measured by the distance between the interface of the TAG lumen – identified by the strong optoacoustic signal from the ink – and the upper boundary of the colon wall blood vessels. Subsequently, the transparent wall of the outer polymer cylinder of the TAG was subtracted.

The mean colon wall thickness was derived by subdividing the pre‐processed volumes into seven equally‐sized segments along the X‐axis and averaging measurements of their respective maximum intensity projections in the YZ‐plane.

*Intensity Measurement*: RSOM signal intensities were measured from segmented colon volumes using ink‐TAG. To reduce artifacts caused by TAG itself, structures outside an angle of ±18° to the TAG‐axis were discarded. Separation of the colon and the overlaying tissue was done manually. For later data analysis with the auto threshold function of Fiji, the resulting volume was set to 8 bits depth and all pixel values were summed up to obtain the total signal intensity, while all pixels different from the colon wall were set to zero.

*Blood Volume Measurement*:The extracted colon volumes were further segmented to obtain the blood volume within the tissue by a threshold calculated using a variation of the IsoData algorithm (Fiji Auto Threshold “Default”). For this, the iterative intermeans^[^ ^33^ ^]^ were calculated based on the histogram of the total volume by disregarding zero values.

### Statistics

Data were analyzed for normal distribution by the Shapiro‐Wilk test prior to further calculations. Single time point measurements (e.g., histology, colon length) were analyzed for group differences (sham d7 vs DSS d9 and sham d7 vs DSS d28) by either the Mann‐Whitney test or unpaired t‐test.

In the mild colitis study, endoscopic scoring and RSOM measurements were analyzed for the following group differences: sham d0 versus sham d7, DSS d7 versus DSS d9, and sham d7 versus DSS d9 by Kruskal‐Wallis test or one‐way ANOVA with multiple comparisons.

In the severe colitis study, endoscopic scoring and RSOM measurements were analyzed in a paired manner between each time point by either the Friedman test or mixed‐effect analysis. Correlations were provided as Spearman correlation coefficient (r_s_). For all calculations two‐tailed *P*‐values < 0.05 were regarded as statistically significant. GraphPad Prism software (Version 9, GraphPad Software, Inc., San Diego, CA, USA) was used for all statistics.

## Conflict of Interest

M.J.W., F.K., and A.P.R. are shared patent holders with iThera medical GmbH (Munich, Germany) on an optoacoustic imaging system/software, however, different from the one described in the study.

## Author Contributions

S.E.B. and A.P.R. contributed equally to the work. A.B., F.K., S.E.B., and A.P.R. designed the study. A.B. developed the TAG. A.B., M.O., and E.B. performed all animal experiments. A.B. performed all imaging analyses. M.E. scored the histopathology data. F.K. scored the endoscopy data. A.B., F.K., S.E.B., and A.P.R. analyzed the data. A.B., E.B., L.P., M.E., M.O., O.T., U.R., A.H., A.H., M.F.N., J.W., O.F., M.J.W., F.K., S.E.B., and A.P.R. interpreted the data. A.B. and A.P.R. wrote the first draft of the manuscript. The manuscript was critically reviewed by all authors.

## Supporting information

Supporting Information

Supplemental Video 1

## Data Availability

The data that support the findings of this study are available from the corresponding author upon reasonable request.
