# Supplementary material for: Transrectal Absorber Guide Raster‐Scanning Optoacoustic Mesoscopy for Label‐Free In Vivo Assessment of Colitis
Source: Adv Sci (Weinh). 2023 Apr 21;10(18):2300564. doi: 10.1002/advs.202300564 (PMC10288266; doi:10.1002/advs.202300564)
Supplement: Supplementary file 1 — Supporting Information [file ADVS-10-2300564-s001.pdf]

ADVANCED SCIENCE
Open Access

Supporting Information

for *Adv. Sci.*, DOI 10.1002/advs.202300564

Transrectal Absorber Guide Raster-Scanning Optoacoustic Mesoscopy for Label-Free In Vivo Assessment of Colitis

Adrian Buehler, Emma Brown, Lars-Philip Paulus, Markus Eckstein, Oana-Maria Thoma, Mariam-Eleni Oraiopoulou, Ulrich Rother, André Hoerning, Arndt Hartmann, Markus F. Neurath, Joachim Woelfle, Oliver Friedrich, Maximilian J. Waldner, Ferdinand Knieling, Sarah E. Bohndiek and Adrian P. Regensburger**

Supporting Information

Transrectal Absorber Guide Raster-Scanning Optoacoustic Mesoscopy for Label-Free *in vivo* Assessment of Colitis

Adrian Buehler, Emma Brown, Lars-Philip Paulus, Markus Eckstein, Oana-Maria Thoma, Mariam-Eleni Oraiopoulou, Ulrich Rother, André Hoerning, Arndt Hartmann, Markus F. Neurath, Joachim Woelfle, Oliver Friedrich, Maximilian J. Waldner, Ferdinand Knieling, Sarah E. Bohndiek and Adrian P. Regensburger **

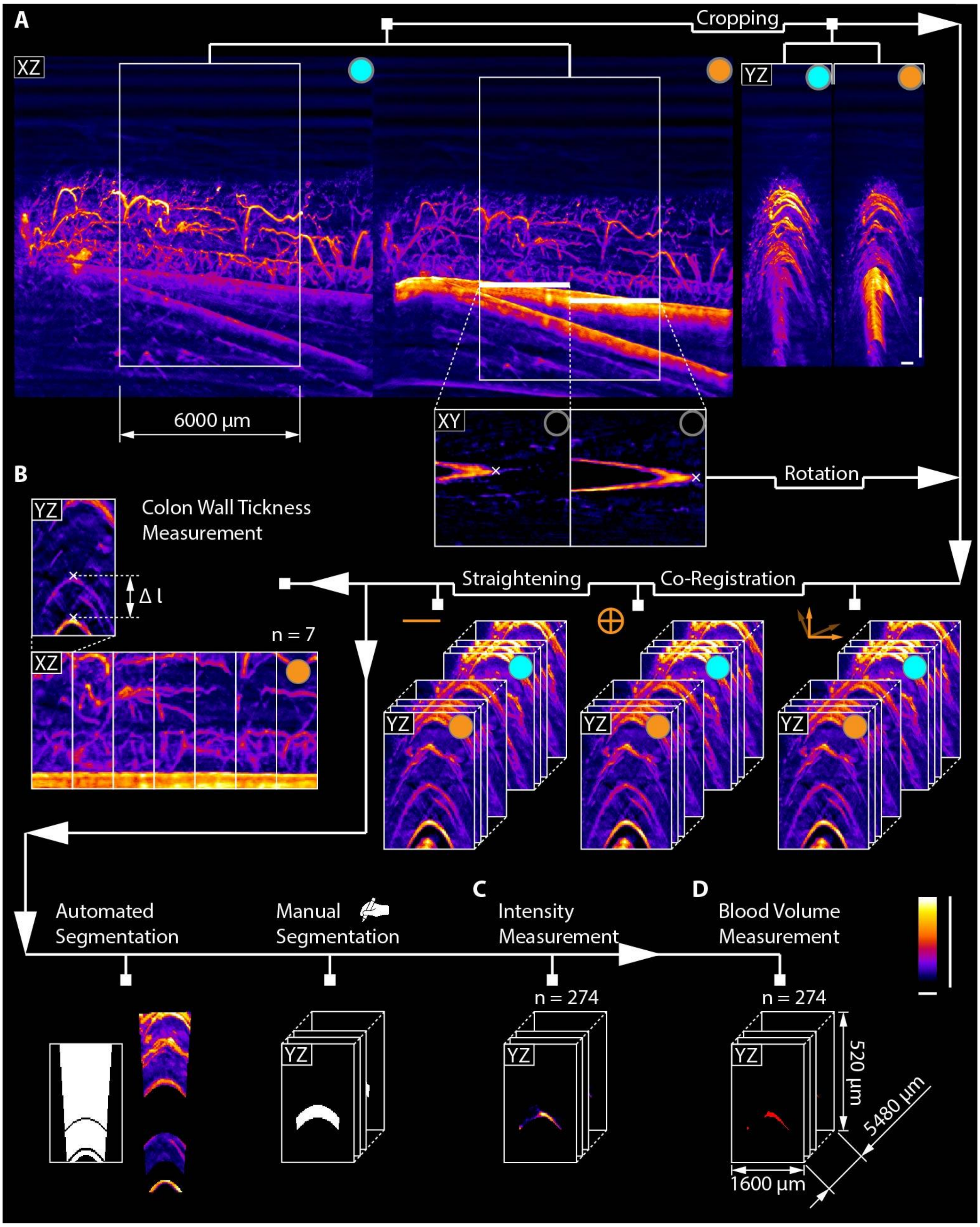

Figure S1. RSOM image co-registration and data processing

A: At each imaging time point, each mouse was scanned two times consecutively without repositioning – with water (blue dot) and with ink (orange dot) filled TAG. A standardized

image section (6000 µm) was further processed by rotation [1], co-registration [2], straightening, automated and manual segmentation.

B: RSOM colon wall thickness was measured in seven maximum intensity projections in the transverse plane of the volume subdivided into seven segments. Therefore, the distance Δl between the colon wall vessels and the interface of the TAG's lumen and its outer polymer cylinder was measured and the constant cylinder wall thickness was subtracted.

C: RSOM signal intensity was calculated as the cumulative sum of voxels of the segmented colon volume inside a ±18° angle relative to the X (TAG) axis.

D: RSOM blood volume was obtained from the number of voxels of the segmented colon volume that were above a threshold calculated on the basis of the whole histogram by the IsoData algorithm.[3]

All scale bars represent 400 µm.

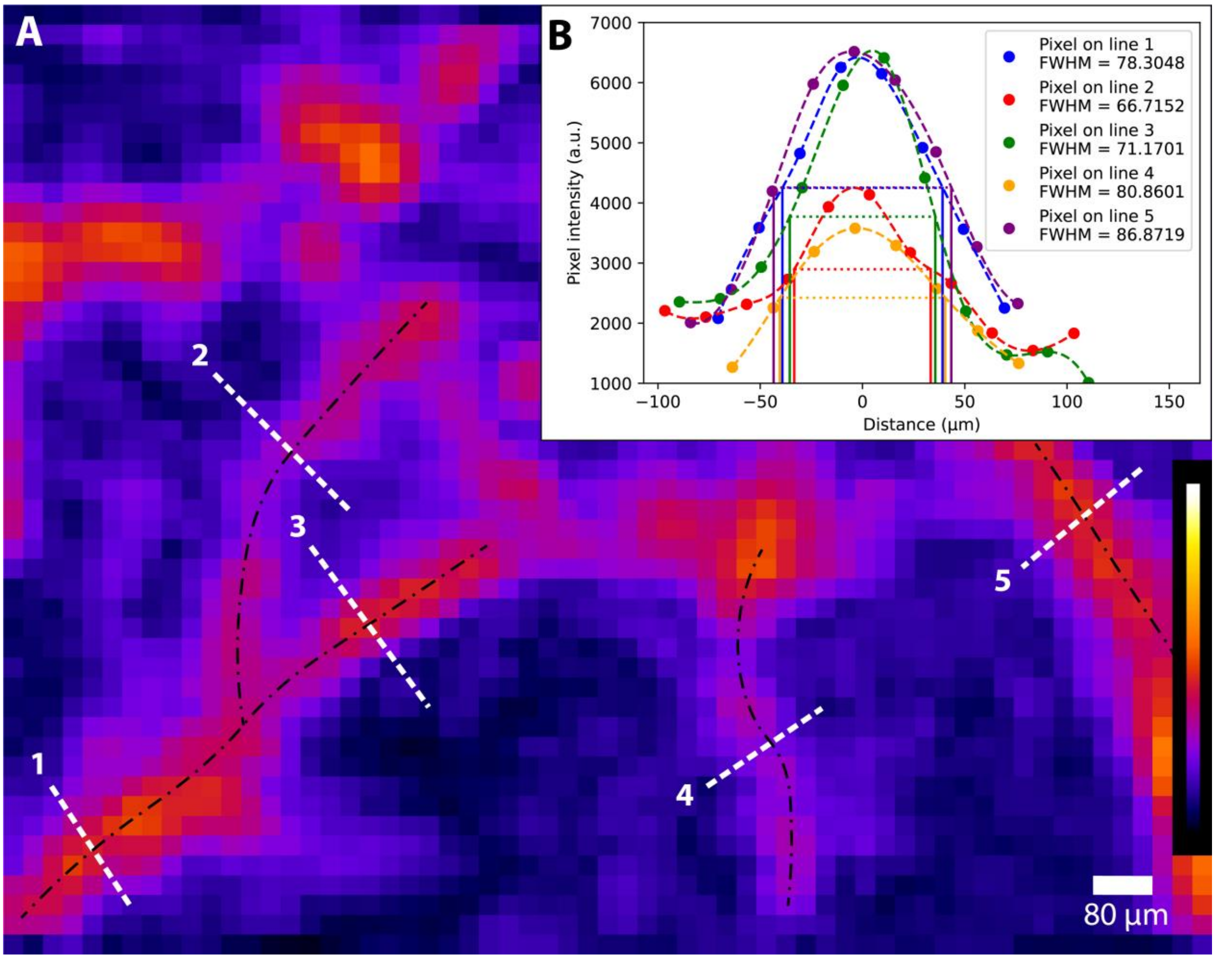

Figure S2. Zoomed section of a maximum intensity projection of the colon wall.

This zoomed section from **Figure 5** shows the colon wall of the first mouse from the longitudinal study at day 7 as a maximum intensity projection in the frontal plane at higher resolution.

A: The blood vessels marked with black dash-dotted lines are clearly distinguishable from the background. For an estimation of the smallest measurable vessels, the full-width half-maximum (FWHM) was calculated at five locations based on the pixel intensities along the white dashed lines.

B: In addition to the intensity values of the individual pixels, the FWHM (full-width visualized by vertical lines; half-maximum visualized by colored dotted lines) was calculated based on cubic interpolation (colored dashed lines). The average FWHM was 76.78 ± 7.96 µm.

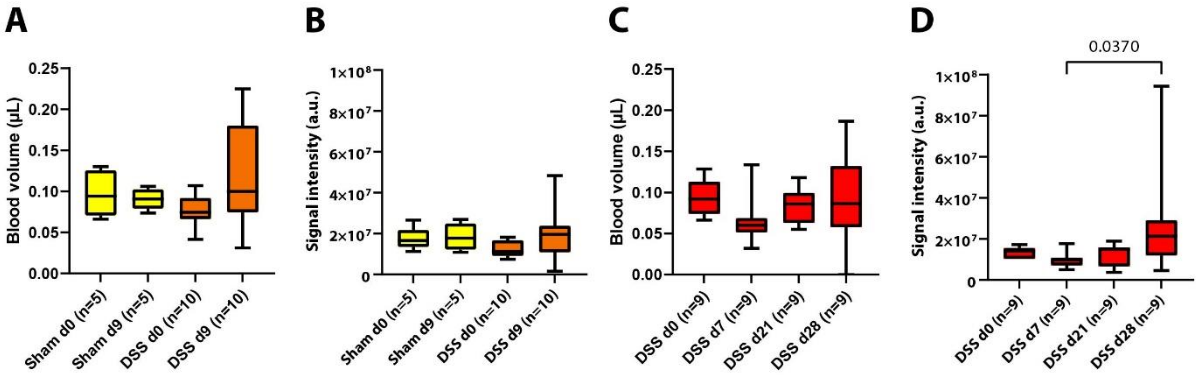

Figure S3. RSOM imaging using water-TAG

The data displayed in this figure was generated using water instead of ink in TAG-RSOM. **A**–**D**: RSOM derived blood volume (**A**) and signal intensities (**B**) in mild colitis group and also blood volume (**C**) and signal intensities (**D**) in severe colitis group suggest a negligible influence of possible artefacts caused by an increased absorption of the ink-TAG.
DSS = dextran sulfate sodium, d = day, TAG = transrectal absorber guide; $P < 0.05$ was considered statistically significant.

Table S1

A: Mild colitis data set

ID	Day	RSOM	Endosc	Histo-1	Histo-2	Disease	Weight	Colon
1	0	Present	Present			Present	Present	
1	9	Present	Present	n=3	n=3	Present	Present	Present
2	0	Present	Present			Present	Present	
2	9	Present	Present	n=3	n=3	Present	Present	Present
3	0	Present	Present			Present	Present	
3	9	Present	Present	n=3	n=3	Present	Present	Present
4	0	Present	Present			Present	Present	
4	9	Present	Present	n=3	n=1	Present	Present	Present
5	0	Present	Present			Present	Present	
5	9	Present	Present	n=2	n=3	Present	Present	Present
6	0	Present	Present			Present	Present	
6	9	Present	Present	n=2	n=2	Present	Present	Present
7	0	Present	Present			Present	Present	
7	9	Present	Present	n=2	n=1	Present	Present	Present
8	0	Present	Present			Present	Present	
8	9	Present	Present	n=1	n=1	Present	Present	Present
9	0	Present	Present			Present	Present	
9	9	Present	Present	n=3	n=2	Present	Present	Present
10	0	Present	Present			Present	Present	
10	9	Present	Present	n=3	n=3	Present	Present	Present

B: Severe colitis data set

ID	Day	RSOM	Endosc	Histo-1	Histo-2	Disease	Weight	Colon
1	0	Present	Present			Present	Present	
1	7	Present	Present			Present	Present	
1	21	Present	Present			Absent	Present	
1	28	Present	Present	n=1	n=1	Present	Present	Present
2	0	Present	Present			Present	Present	
2	7	Present	Present			Present	Present	
2	21	Present	Present			Absent	Present	
2	28	Present	Present	n=3	n=1	Present	Present	Present
3	0	Present	Present			Present	Present	
3	7	Present	Present			Present	Present	
3	21	Present	Present			Absent	Present	
3	28	Present	Present	n=3	n=1	Present	Present	Present
4	0	Present	Present			Present	Present	
4	7	Present	Present			Present	Present	
4	21	Present	Present			Absent	Present	
4	28	Present	Present	n=2	n=3	Present	Present	Present
5	0	Present	Present			Present	Present	
5	7	Present	Present			Present	Present	
5	21	Present	Absent			Absent	Present	
5	28	Present	Present	n=2	n=2	Present	Present	Present
6	0	Present	Present			Present	Present	
6	7	Present	Present			Present	Present	
6	21	Present	Present			Absent	Present	
6	28	Present	Present	n=1	n=3	Present	Present	Present
7	0	Present	Present			Present	Present	
7	7	Present	Present			Present	Present	
7	21	Present	Present			Absent	Present	
7	28	Present	Present	n=3	n=2	Present	Present	Present
8	0	Present	Present			Present	Present	
8	7	Present	Present			Present	Present	
8	21	Present	Present			Absent	Present	
8	28	Present	Present	n=2	n=1	Present	Present	Present
9	0	Present	Present			Present	Present	
9	7	Present	Present			Present	Present	
9	21	Present	Absent			Absent	Present	
9	28	Present	Present	n=3	n=2	Present	Present	Present
10	0	Present	Present			Present	Present	
10	7	Present	Present			Present	Present	
10	21	Present	Absent			Absent	Present	
10	28	Absent	Absent	Absent	Absent	Absent	Absent	Absent

C: Sham control data set

ID	Day	RSOM	Endosc	Histo-1	Histo-2	Disease	Weight	Colon
1	0	Present	Present			Absent	Present	
1	7	Present	Present	n=3	n=3	Absent	Present	Present
2	0	Present	Present			Absent	Present	
2	7	Present	Present	n=3	n=3	Absent	Present	Absent
3	0	Present	Present			Absent	Present	
3	7	Present	Present	n=3	n=1	Absent	Present	Present
4	0	Present	Present			Absent	Present	
4	7	Present	Present	n=3	n=1	Absent	Present	Present
5	0	Present	Present			Absent	Present	
5	7	Present	Present	n=3	n=3	Absent	Present	Present

Table S1. Overview of data sets

The table shows the completeness of the data set mild colitis in **A**, severe colitis in **B**, and sham in **C,** for each mouse (ID) and imaging time point (Day). It includes information on the success of TAG-RSOM imaging and thus on the subsequent analysis of blood volume, colon wall thickness, and signal intensity. The histological scoring was based on a *n* native colon tissue sections, and the evaluation of colon wall thickness was determined by averaging four measurements on each of *n* stretched colon tissue sections (as shown in **Figure 3E**). Histo-1 = native, standard histology, Histo-2 = stretched histology

Supplementary References

[1] E. H. Meijering, W. J. Niessen, M. A. Viergever, *Med Image Anal* **2001**, *5* (2), 111, https://doi.org/10.1016/s1361-8415(00)00040-2.
[2] R. Fernandez, C. Moisy, *Bioinformatics* **2021**, *37* (10), 1482, https://doi.org/10.1093/bioinformatics/btaa846.
[3] T. W. Ridler, S. Calvard, *Ieee T Syst Man Cyb* **1978**, *8* (8), 630, https://doi.org/DOI 10.1109/tsmc.1978.4310039.
